# A Tuneable Switch for Controlling Environmental Degradation of Bioplastics: Addition of Isothiazolinone to Polyhydroxyalkanoates

**DOI:** 10.1371/journal.pone.0075817

**Published:** 2013-10-11

**Authors:** Catherine Anne Woolnough, Lachlan Hartley Yee, Timothy Stuart Charlton, Leslie John Ray Foster

**Affiliations:** 1 Bio/Polymer Research Group and Centre for Advanced Macromolecular Design, School of Biotechnology and Biomolecular Sciences, University of New South Wales, Sydney, New South Wales, Australia; 2 Marine Ecology Research Centre, School of Environment, Science and Engineering, Southern Cross University, Lismore, New South Wales, Australia; 3 Centre for Marine Bio-Innovation, University of New South Wales, Sydney, New South Wales, Australia; Brandeis University, United States of America

## Abstract

Controlling the environmental degradation of polyhydroxybutyrate (PHB) and polyhydroxyvalerate (P(HB-co-HV)) bioplastics would expand the range of their potential applications. Combining PHB and P(HB-co-HV) films with the anti-fouling agent 4,5-dichloro-2-n-octyl-4-isothiazolin-3-one (DCOI, <10% w/w) restricted microbial colonisation in soil, but did not significantly affect melting temperature or the tensile strength of films. DCOI films showed reduced biofouling and postponed the onset of weight loss by up to 100 days, a 10-fold increase compared to unmodified films where the microbial coverage was significant. In addition, the rate of PHA-DCOI weight loss, post-onset, reduced by about 150%; in contrast a recorded weight loss of only 0.05% per day for P(HB-co-HV) with a 10% DCOI loading was observed. This is in stark contrast to the unmodified PHB film, where a recorded weight loss of only 0.75% per day was made. The ‘switch’ that initiates film weight loss, and its subsequent reduced rate, depended on the DCOI loading to control biofouling. The control of biofouling and environmental degradation for these DCOI modified bioplastics increases their potential use in biodegradable applications.

## Introduction

On our planet of finite resources, the need to employ practises which encourage reuse, recycling and a return of the resources to natural cycles, becomes an essential and sustainable goal. The use of synthetic polymers and their subsequent disposal has produced a scourge of material incapable of degrading or composting within reasonable time periods. Thus, the use of degradable or compostable polymers forms the basis of best practise with the environment’s health in mind. Polymers capable of degrading can be broken down into the following categories: degradable, compostable and biodegradable [1]. Polyhydroxyalkanoates (PHAs) are one of the most recognised biodegradable plastics producing zero toxic waste and capable of recycling completely into recyclable organic matter [1], [2].

The methods in which polymers degrade or compost are varied and wide, where the catalysts for degradation include UV light [3], heat [4] and either aerobic [5] or anaerobic environments that select for certain bacterial species [6]. The bacteria themselves, breakdown polymers by producing depolymerase enzymes [2]. Our study focuses on bacterially driven degradation of polyhydroxyalkanoate polymers and demonstrates by inhibiting bacterial processes using an antifouling compound such as 4,5-dichloro-2-*n*-octyl-4-isothiazolin-3-one (DCOI), the degradation rate for PHA and likely biodegradable polymers in general can be ‘tuned' using varying concentrations of added DCOI.

We have previously reported that a number of these so-called ‘green plastics’ fail to demonstrate environmental degradation and would, perhaps, be more appropriately classified as ‘compostable’ [7]. In contrast, microbial short chain length polyhydroxyalkanoates (*scl*-PHAs) such as poly(3-hydroxybutyrate); PHB and poly(3-hydroxybutyrate*-co-*3-hydroxyvalerate); P(HB*-co-*HV), are readily degraded in a range of natural environments including soils [7], [8], anaerobic and aerobic sludge, composts [9] and marine as well as fresh waters [10]. Environmental degradation of PHAs proceeds via a combination of abiotic and enzymatic hydrolysis, with the latter mechanism having the greatest influence [10], [11]. However, this propensity for biodegradation in the environment also restricts their potential environmental applications and a postponement of environmental degradation may be useful for applications such as garbage bags, potting containers, building materials and food packaging, agricultural sheets used to protect young plants and coatings for fish nets.

The current proposed methods for controlling environmental degradation of polymers have included altering polymer composition, side chains, molecular weight or crystallinity [12]-[16]. However, these techniques also change the material properties and it has been demonstrated that a significant relationship between the biofouling of bioplastics and their environmental degradation exists, particularly with surface rugosity, a major influence on microbial colonisation [7], [11]. By logical extension it is easy to postulate that control of biofouling will affect the environmental degradation of biodegradable plastics.

Historically, the most common antifouling strategies involved tin based additives to coatings, such as organotin (e.g.tributyltin) to paint [17], which although effective at preventing biofouling, are toxic to non-target organisms, bioaccumulate within the environment and pose a significant health risk. Consequently, the International Maritime Organization banned all organotin compounds in 1999 (IMO Resolution A. 895 21, 25/11/1999). Copper is now widely used as an antifoulant. However copper also has a high degree of toxicity towards non-target organisms [18], [19].

**Figure 1:**
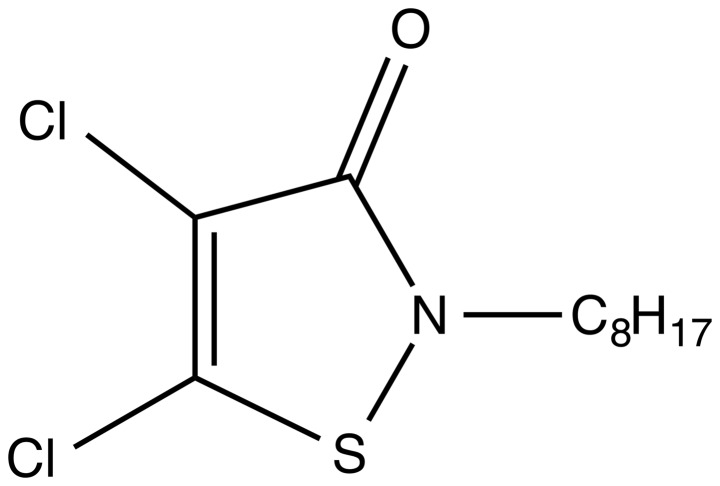
Chemical structure of the antifouling agent 4,5-dichloro-2-n-octyl-4-isothiazolin-3-one, (DCOI).

In contrast to the metal-based antifoulants, 4,5-dichloro-2-*n*-octyl-4-isothiazolin-3-one DCOI (Figure 1) is an anti-fouling agent that biodegrades to a non-halogenated open-ringed product, which is reported to be five times less toxic than the parent compound. In addition, the degradation rate is comparatively rapid via a chemically unstable N-S bond in the isothiazolone ring [20] and resulting metabolites become strongly bound to sediment or soil particles, thereby immobilising them and greatly reducing bioavailability [21].

The blending of hydrophobic PHAs with hydrophobic additives is reported to result in a homogeneous mix [22], [23]. Hence, because DCOI is a hydrophobic additive [24], it is therefore an excellent candidate for blending with the hydrophobic PHA biopolymers. Consequently, it is proposed that this blend will result in bioplastics with a sustained release of DCOI that will prevent biofouling and degradation of these bioplastics in environmental applications, particularly where exposure to biofouling and degrading organisms is high.

## Materials and Methods

### Materials and reagents

Polyhydroxybutyrate, PHB (M_w_ = 600,000 g mol^−1^) and P(HB*-co-*8HV) with 8 mol% 3-hydroxyvalerate units, (M_w_ = 252,000 g mol^−1^) were obtained from Sigma-Aldrich (Sydney, Australia, batch numbers 27331CS, 12703MD respectively). DCOI (4,5-dichloro-2-*n*-octyl-4-isothiazolin-3-one 211) was obtained from Rohm and Haas, (Philadelphia, USA). All other chemicals used were obtained from APS Chemicals (Seven Hills, NSW, Australia) and were of analytical grade and used as obtained.

### Polymer film fabrication

PHB and P(HB-*co*-8HV) were dissolved in chloroform (60°C) at 4.5% (w/v) and fabricated into films by casting into glass Petri dishes, covered and allowed to evaporate (96 h, 25°C, relative humidity: 30%). Films were subsequently removed and dried under vacuum (48 h, 25°C) before standing for a further 24 h (25°C, RH = 30%) until their weights had atmospherically equilibrated. Films were then annealed for three weeks before being cut into square samples (1 cm^2^, 200±10 µm thick, 20±3 mg, microgram balance Cahn C-33, Orion, USA, precision: ±10 µg). Films containing DCOI were fabricated as described above with the DCOI dissolved in chloroform before addition to the dissolved PHA solution and stirred (25°C, 2 h, 200 rpm) at loadings of 2.5, 5 and 10% (w/w).

### Differential scanning calorimetry

The influence of DCOI on the thermal properties of PHB and P(HB-*co-*8HV) were measured using a Perkin Elmer DSC 7 calorimeter equipped with a TAC 7/DX thermal analysis controller, a CCA 7 controlled cooling accessory and a DPA 7 photo calorimeter as previously described [25]. To eliminate thermal history, samples were initially heated from −10 to 220°C at 10°C min^−1^ (1^st^ heating run), held for 1 min then cooled to −10°C at the same rate (1^st^ cooling run). Samples were then run a second time using the same heating and cooling parameters as in the first cycle, where the melting and crystallisation temperatures (T_m_ and T_c_) were obtained; samples were run in triplicate (*n* = 3).

### Material properties

Films were examined using a calibrated tensiometer (Instron Mini 5543, MA, USA). Samples (10×20 mm, 200±10 µm thick) were secured to the tensiometer using pneumatic grips, which moved apart at 1 mm.min^−1^ until the polymer films failed. The tensile strength and elongation required to cause failure were measured using BlueHills software (*n* = 10, Instron, MA, USA).

### Monitoring environmental degradation

Environmental degradation of the bioplastics samples was conducted in “mature soil” under controlled conditions within, the University of New South Wales, Sydney, Australia glasshouse. All soil materials were sieved to less than 2 mm in diameter and mixed thoroughly before polymer film burial (ASTM D 5988). Polymer films were randomly buried 2 cm apart at 15±5 cm below the soil surface in a 1×2 m plot. The spatial arrangement of the polymer films ensured that total polymer weight did not exceed 7.7% (w/w) of the soil (ASTM D 6003). Polymer films were removed periodically for weight loss and biofilm formation measurements as previously described [7], [11]. Soil temperatures ranged from 11°C (night) to 30°C (day), soil pH was 6.7, and soil moisture was maintained between 17 and 23%. Five-polymer film replicate samples were removed at each sampling period during the 150-day burial trial (*n* = 5).

Once removed from the soil, the soil particulates and any biofilms attached to the polymer film surfaces were removed by sonication in a solution containing 0.25% sodium hypochlorite, 0.1% Tween-85 and 0.01% Savinase Ultra (Novozyme, Australia). Films were then dried in a vacuum dessicator (96 h, 35°C) before being removed and allowed to equilibrate within air (48 h, 25°C, RH = 30%) prior to a determination of sample weight (Cahn C-33, Orion, USA, accuracy: ±10 µg); losses of DCOI from the washing procedure were negligible.

### DCOI loading measurements

To confirm distribution of DCOI within the PHB and P(HB-*co*-8HV) films, five (*n* = 5) random 8×8 mm pieces were cut from circular films with diameters of 7.5 cm and submerged in ethyl acetate. DCOI loading measurements were made by repeatedly immersing in fresh samples of ethyl acetate for 30 min periods until the spectrophotometric absorbance (286 nm) of the final solution was less than 0.01 (Shimadzu UV-Vis 160, Japan). The DCOI concentration was determined using a standard curve containing DCOI dissolved in ethyl acetate.

### Adhered biofilm monitoring

Polymer film samples removed from the soil were vortexed for 5 s in reverse osmosis (RO) water to remove non-adhered cells. Films were then stained with SYTO®, 9 nucleic acid stain (fluorescent nucleic acid stain, Molecular Probe Inc., Eugene, USA) for imaging of the adhered biofilms on the polymer surfaces using a Leica confocal laser scanning microscope (CLSM, Leica TCS SP Confocal DMIRB, Germany; argon laser excitation 488 nm, emission wavelength 520 – 550 nm) [7], [11]. A mean biofilm surface coverage was determined as an average of three images from each side of three replicate polymer films (*n* = 3×6) where the biofilm and polymer surface were recorded through the *z* plane (step size: 0.5 µm). Obtained images were analysed using Adobe Photoshop® and the percentage surface area covered by biofilm determined through quantifiying pixels [26].

### Surface roughness analysis

Surface rugosity (as average surface roughness, *R*_a_) was quantitatively determined for both undegraded and partially degraded films as described previously [7], [11]. Film surfaces were imaged in reflection mode of a confocal laser scanning microscope (excitation 458 nm, emission 440 − 470 nm). Multiple images through the *z* plane (step size  = 0.5 µm) were recorded. The average surface roughness (*R*_a_) was calculated as a mean from 10 images per side of each film using the ImageJ software (National Institutes of Health, USA, *n* = 10) according to ISO 4298 using equation 1, [27], [28]:
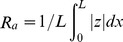
(1)


where ‘*L*’ is the sampling length, ‘*z*’ the plane and ‘*dx*’ the variations of irregularities from the mean line.

Polymer films were examined under a Scanning Electron Microscope at magnifications of 1000x, 2500x and 5000x at 10.0 kV with a spot size of 4.0, with an FEI Quanta 200 Electron Microscope, (Brunswick, USA). PHA films were gold coated and partially degraded polymer films had any biofilms removed (see below) and dried prior to being gold coated.

## Results and Discussion

### Physical properties

Both the *short chain length-*Polyhydroxyalkanoates (*scl-*PHAs) used in this study and the DCOI antifoulants are hydrophobic and readily dissolved in the common solvent chloroform. In addition, a homogenous distribution of DCOI throughout the PHB and P(HB-*co*-8HV) films is preferred to provide a consistent release of DCOI. The DCOI concentrations determined through absorbance at 286 nm were within one standard deviation, 0.3% (w/w), of the calculated loading for the polymer films. Consequently, DCOI was readily blended and evenly distributed with these *scl-*PHAs, however blending to form composites may alter the properties of these materials. Hence, as these *scl-*PHAs are classified as thermoplastics [29], the thermal and material properties of these PHA-DCOI composites are of particular importance for their potential industrial processing and application as bioplastics.

**Figure 2:**
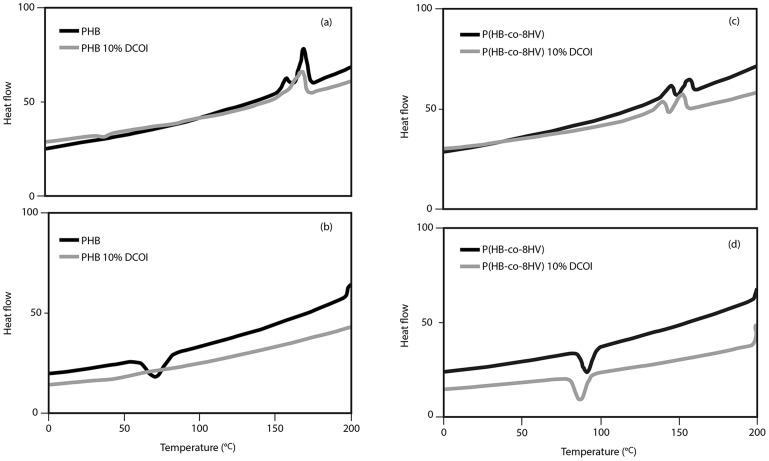
Differential Scanning Calorimetry curves of virgin PHB films and P(HB-*co*-8HV) films (black line) and films containing 10% DCOI (grey line) in the second post annealed heating (a,c) and cooling (b,d) runs respectively.

DSC thermograms for the second heating and cooling experiments of the PHA-DCOI composites, (Figure 2), shows melting peaks of 169 and 157°C for PHB and P(HB-co-8HV) respectively, which were consistent with previous reports [25], [29]. However, addition of DCOI at concentrations of 5 and 10% (w/w) lowered these melting point temperature (T_m_) peaks by up to 5°C, but at DCOI concentrations of 2.5% (w/w), there was negligible change in either melting point or crystallisation temperatures (Table 1). PHB re-crystallisation from the molten state occurred at 71°C (T_c_) (Figure 2b), although the addition of DCOI influenced re-crystallisation giving a comparatively broader T_c_ valley for PHB with 5% (w/w) DCOI loading and a reduction in T_c_ to 61°C (Table 1). Increasing the antifoulant loading to 10% (w/w) DCOI prevented re-crystallisation (Figure 2b). This trend is consistent with reports of PHB blended with similar compounds such as poly(ethylene glycol) or poly(vinyl alcohol) where re-crystallisation is retarded [30], [31]; crystallisation temperatures for P(HB-*co*-8HV)-DCOI composites were seen, but up to 4°C lower (Figure 2d, Table 1).

**Table 1:** Melting point (T_m_) and crystallisation temperature (T_c_) with loading of DCOI in *scl*-PHA films.

	Melting (°C)	Crystalization (°C)
**PHB**	169	71
**PHB 2.5% DCOI**	169	70
**PHB 5.0% DCOI**	168	61
**PHB 10% DCOI**	167	-
**P(HB-** ***co*** **-8HV)**	157	88
**P(HB-** ***co*** **-8HV) 2.5% DCOI**	157	88
**P(HB-** ***co*** **-8HV) 5.0% DCOI**	155	87
**P(HB-** ***co*** **-8HV) 10% DCOI**	152	84

The tensile strength of the films was also investigated and no discernable changes in tensile strength were observed as a consequence of DCOI loading. Rather, P(HB-*co*-8HV) and the DCOI composites showed tensile strengths of around 7±0.9 Mpa, whereas PHB-DCOI films had tensile strengths of 13±1.3 MPa. In addition, there were small linear increases (R^2^ = 0.99 for PHB and 0.97 for P(HB-*co*-8HV)) for measured extension to break lengths, which increased by about 1.5% for both polymers with increasing DCOI additions.

### Degradation and the effect of DCOI loading

**Figure 3:**
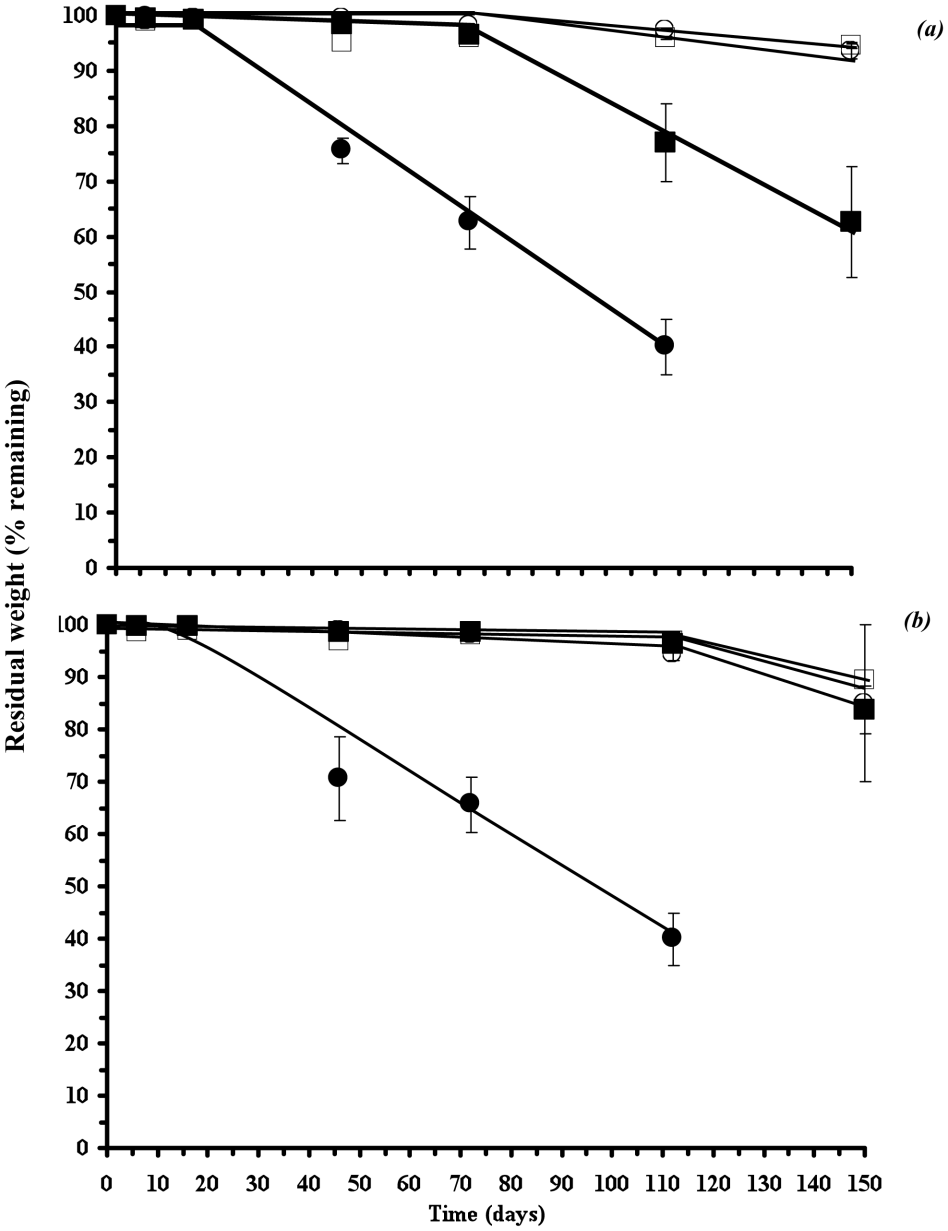
Residual weight of PHB-DCOI (a) and P(HB-co-8HV)-DCOI films buried in soil; (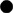) 0% w/w, (▪) 2.5%, (○) 5% and (□) 10% (w/w) initial DCOI loadings.

After soil burial trials, both the PHB and P(HB-*co*-8HV) films showed negligible weight loss during the first 16 days of environmental exposure, after which they lost weight at an approximately linear rate of 0.75% per day (Figure 3). Mergaert et al. [8] have reported the degradation of PHB films in soils as ranging from 0.03 to 0.64% per day depending on the soil type, and the findings here were consistent if not slightly higher than those previously reported [8]. Consequently, after 112 days burial in soil, the 0% DCOI loaded PHA films had lost over 60% of their initial weight, however, in contrast, PHB films with a DCOI loading of 2.5% (w/w) showed no significant weight loss until 72 days burial, where films subsequently lost weight at approximately 0.5% per day; again consistent with previous work [8]. This reduced rate of degradation for the PHB-DCOI films compared to their unblended counterparts, suggests that not all the DCOI was released from the films (Figure 3).

**Figure 4:**
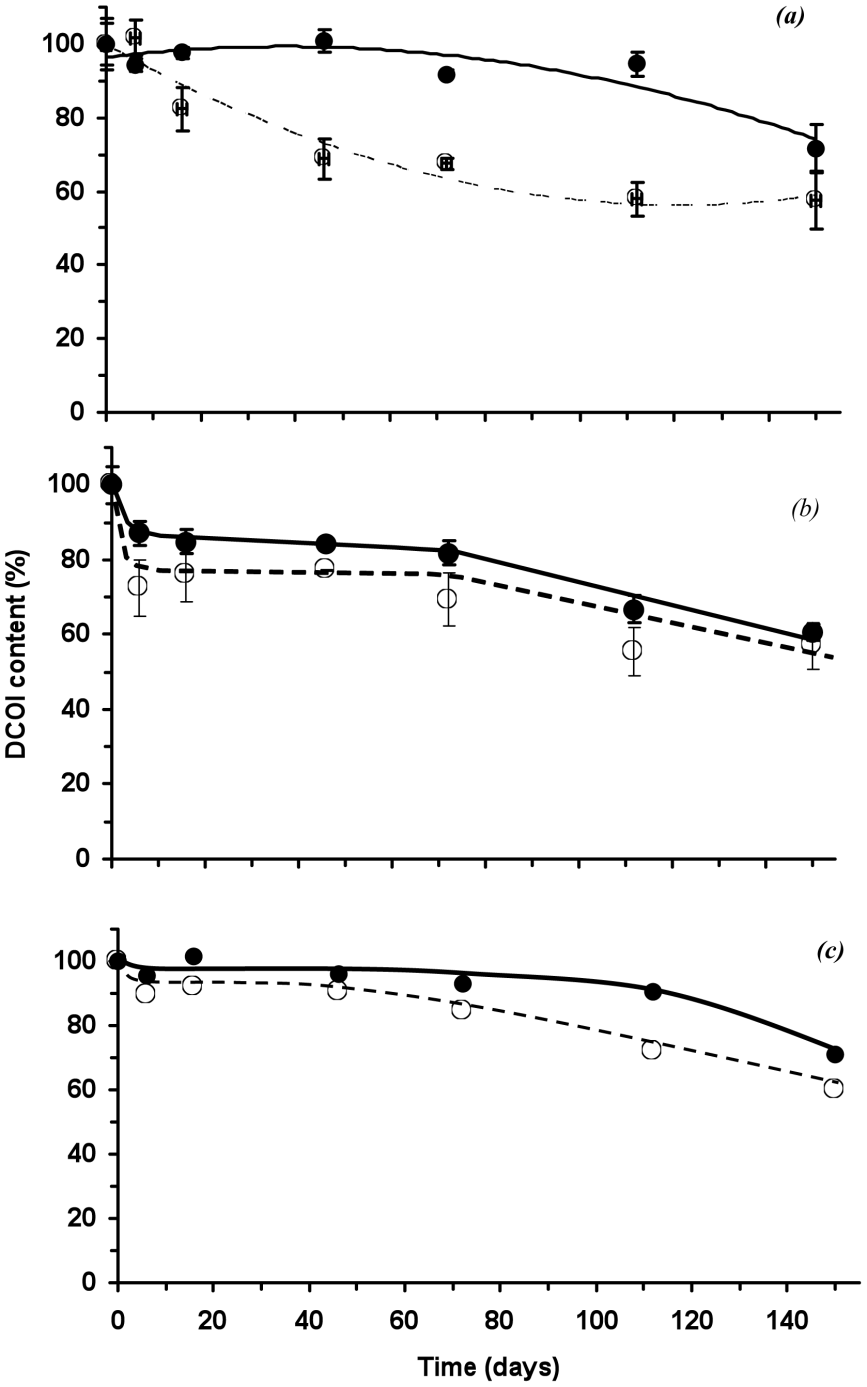
DCOI content in PHB (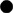) and P(HB-co-8HV) (○ ) films with burial time, (a) 2.5%, (b) 5% and (c) 10% (w/w) initial DCOI loadings.

**Figure 5:**
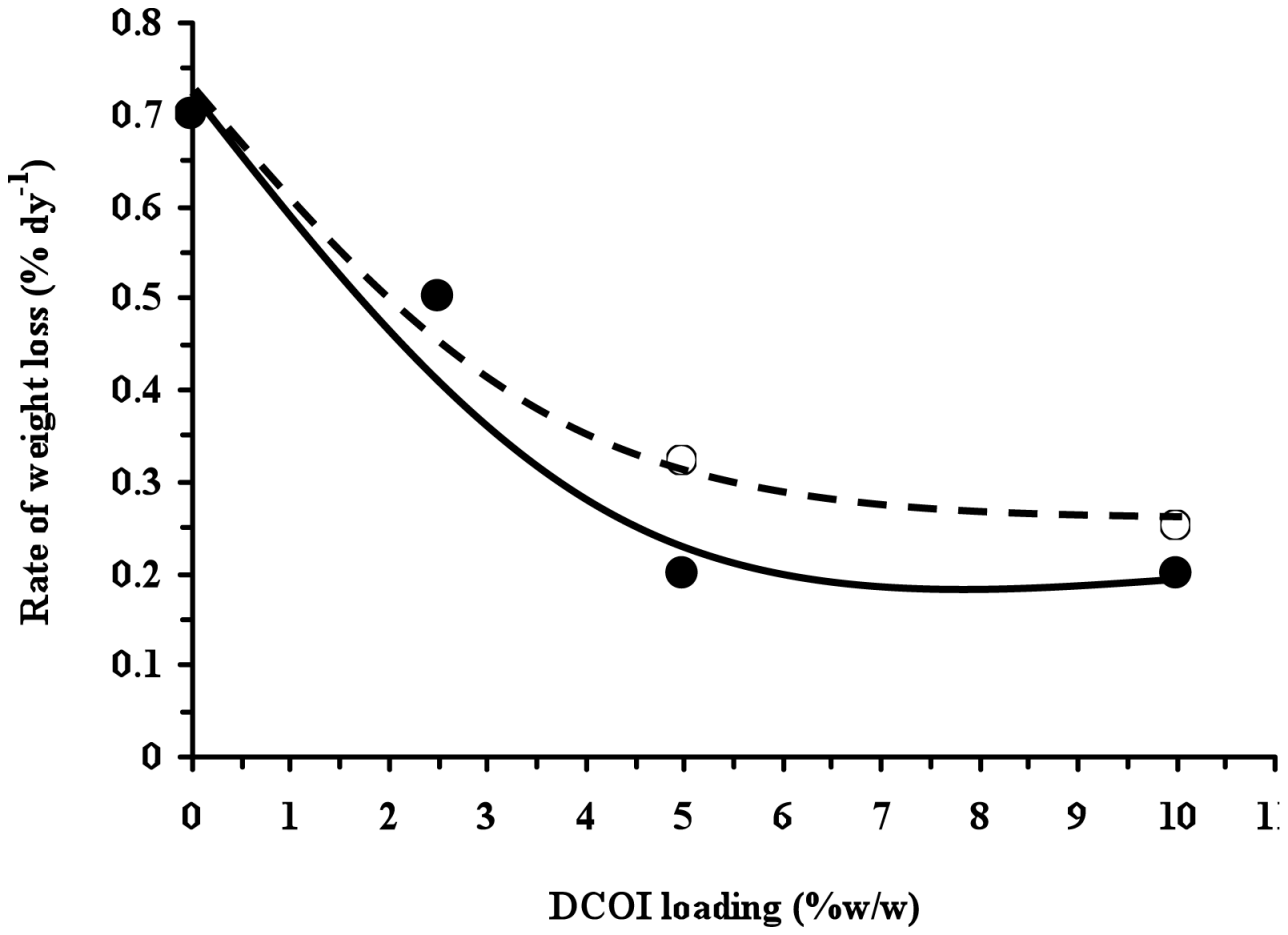
Weight loss rates after initiation of weight loss during burial in mature soil for PHB (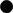) and P(HB-*co*-8HV) (○) films as a consequence of initial DCOI loadings.

This is further supported by Figure 4a, which shows that for the (2.5% w/w) PHB films, DCOI was detected at significant concentrations even after 112 days burial, with over 90% of the antifouling agent still present. Consequently, the post 112 days burial DCOI loss appears directly related to material weight loss observed (Figure 5). In contrast, approximately 40% of the (2.5% w/w) DCOI was released from the P(HB-*co*-8HV) films within the first 72 days of burial, during which time the films exhibited no weight loss (Figure 4a). This suggests that DCOI at initial loadings of 2.5% (w/w) is comparatively less trapped within the P(HB-*co*-8HV) copolymer crystalline matrix than within the homopolymer PHB. However, this initial release of DCOI from P(HB-*co*-8HV) films postpones weight loss of the films for some 112 days of burial, after which the films lost approximately 13% of their weight during the next 38 days (0.33% weight loss per day); a similar rate to PHB-DCOI counterparts (Figure 5).

Increasing the loading of DCOI in PHA films from 2.5 to 5% and 10% (w/w) resulted in an initial release of the antifoulant within the first 9 days of burial. During this initial release period, the PHB 5% DCOI films lost approximately 12% of their DCOI loadings, whereas the P(HB-*co*-8HV) 5% DCOI films lost roughly 23%. These observations further support that a significant proportion of DCOI was less retained in the P(HB-*co*-8HV) copolymer matrix than for the PHB homopolymer (Figure 4b). After this initial burial period, DCOI levels were relatively constant until 72 days burial, after which they declined slowly and this was followed by the onset of weight loss for both 5 and 10% DCOI films from 112 days (Figures 3 and 4). Weight loss of the P(HB-*co*-8HV) DCOI 5 and 10% copolymer films, once initiated, proceeded at comparatively faster rates than the PHB DCOI films, which may be due to abiotic action against the more amorphous copolymer PHA, 0.28%/day for P(HB-*co*-8HV) compared to 0.20%/day for PHB (Figure 5) [7].

Hence, the addition of the antifoulant not only postponed the onset of PHA-DCOI film degradation, as monitored by weight loss (Figure 3), but also subsequently reduced the rates of weight loss (Figure 5). In addition, as DCOI loading increased, the rate of weight loss, also decreased once initiated, and the weight loss rates for PHB-DCOI films were lower than those of the P(HB-*co*-8HV)-DCOI films (Figure 5).

Paints containing antifouling additives, can release the antifouling component in waters either at a linear rate, or rapidly before attaining a steady state of release depending on the chemical nature of the antifouling agent [32]. In our study, the average DCOI leaching rates after 150 days burial were calculated to be 4.1, 9.5 and 15.9 µg cm^−2^ day^−1^ for polymer films originally containing 2.5%, 5% and 10% (w/w) DCOI respectively. DCOI release from paint on boats has been reported as 2 µg cm^−2^ day^−1^ [33] and the manufacturer Rohm and Haas recommends that DCOI application rates of 1-3% (w/w) be used. Compared to the lowest concentration of 2.5% w/w application in our study, DCOI release is comparatively greater from PHAs in soil than from paint in water [33].

### Microbial biofilm coverage and surface roughness

**Figure 6:**
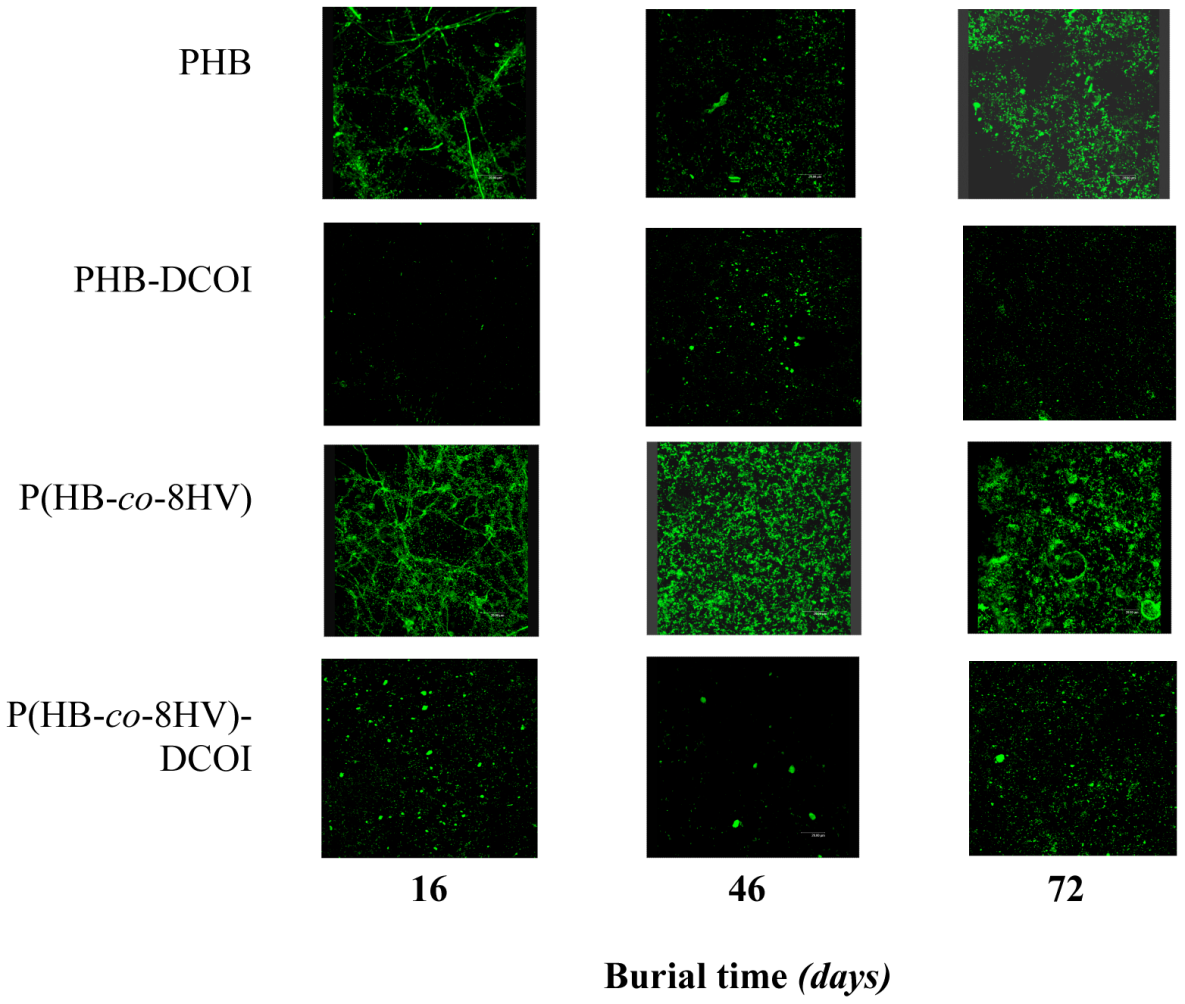
Fluorescent micrographs illustrating comparatively greater microbial colonisation of *scl-*PHA films compared to their DCOI loaded counterparts (2.5% w/w) after burial in mature soil.

**Figure 7:**
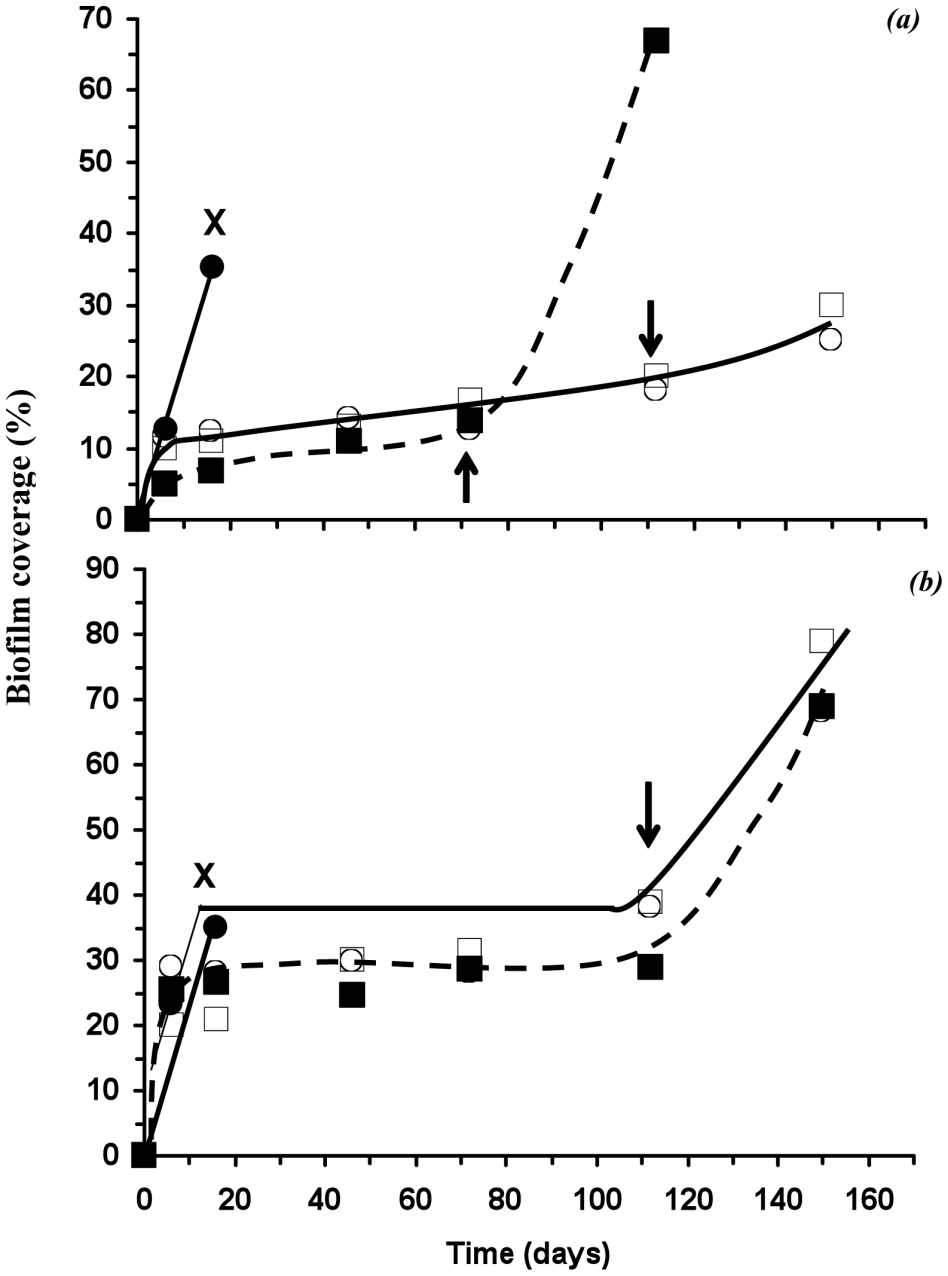
Microbial percent surface area of PHB (a) and P(HB-*co*-8HV) (b) films, with burial time; (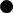) 0% w/w, (▪) 2.5%, (○) 5% and (□) 10% (w/w) DCOI loadings, ‘X’ material weight loss too great to accurately measure biofilm coverage, arrows indicate onset of material weight loss for *scl-*PHA-DCOI films.

Our previous work [7], [11] demonstrated that the microbial and environmental degradation of *scl-*PHA films is positively correlated to surface coverage by micro-organisms. The study reported here is consistent with our previous work concluding the postponement of weight loss onset and the reduced weight loss rates observed was a consequence of reduced biofouling. Furthermore, in our study reported here, fluorescent microscopy showed the surface area of microbiota was the greatest for samples without DCOI (Figure 6). Additionally, the quantitative monitoring of the biofilm coverage showed an initial colonisation of the PHA films, within the first 9 days of burial (Figure 7). However, although colonisation increased until material weight loss began for the unblended PHA films (‘X’ in Figures 7a, 7b), films possessing the DCOI antifoulant showed negligible increased biofilm coverage until significant weight loss occurred (arrows in Figures 7a, 7b, see also Figure 3). PHB-DCOI biofilm coverage remained around 15% while P(HB-*co*-8HV)-DCOI remained around 30% until the onset of weight loss began, after which coverage increased dramatically (arrows in Figures 7a, 7b).

Significantly, the increase in biofilm coverage (Figure 7) corresponds well with the increasing residual weight loss (Figure 3) further reinforcing the link to microbially driven degradation.

PHAs can act as sources of microbial nutrients that support microbial colonisation, the biofouling is not restricted to micro-organisms capable of degrading these polymers. Our previous study [7] reported a direct correlation between PHA weight loss and the microbial populations loosely adhering to the polymer, while the more firmly attached population are apparently opportunists [7].

**Figure 8:**
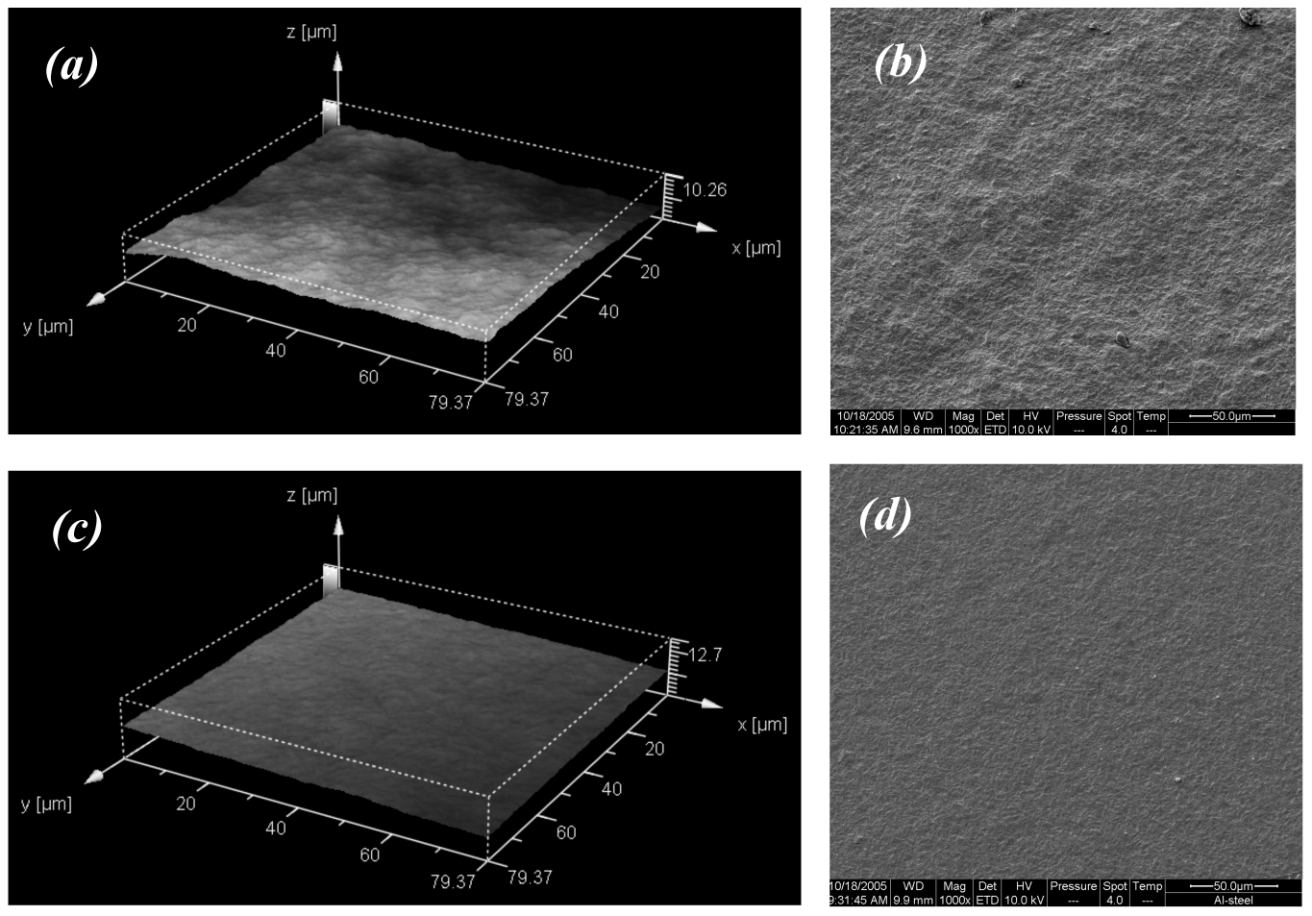
Confocal Laser Scanning Microscopy microtopography and SEM images of undegraded PHB (a, b) and P(HB-*co*-8HV) films (c, d) illustrating surface roughness.

**Figure 9:**
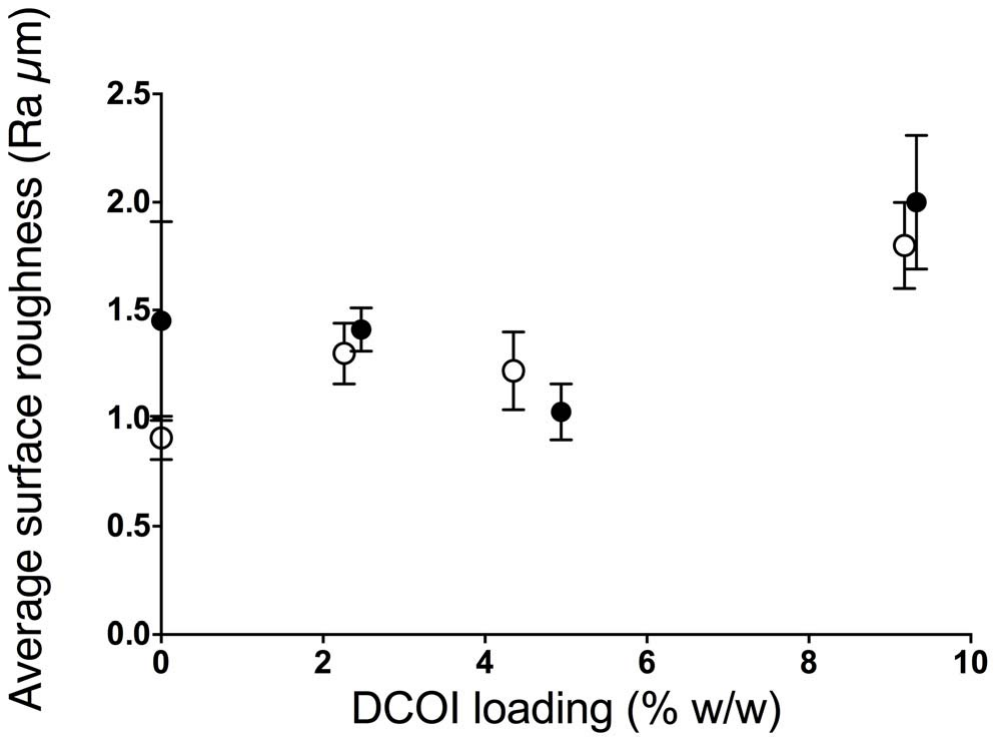
Average Surface Roughness (*R*_a_) for PHB (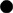) and P(HB-*co*-8HV) (○) films with DCOI loadings.

Biofouling is strongly influenced by surface phenomenon, particularly the microroughness of surfaces, which readily influences microbial attachment [32]. In this study, SEM images show that the undegraded P(HB-*co*-8HV) films were less pitted than PHB counterparts (Figures 8b and 8d); microtopographies of these surfaces further support this observation (Figures 8a and 8c). Depth maps derived from the microtopographic images used to determine the average surface roughness (*R*_a_) provided quantitative data that confirmed the greater rugosity for the PHB film (*R*_a_ = 1.25 µm) compared to P(HB-*co*-8HV) (*R*_a_ = 0.9 µm), (Figure 9). In addition, blending DCOI with PHB had little effect on *R*_a_ until 10% (w/w) where the *R*_a_ increased to 2.0 µm; no crystallisation temperature could be determined for this sample (Figure 9, Table 1). In contrast, blending DCOI with P(HB-*co*-8HV) showed an apparently linear increase in *R*_a_ similar to its PHB-DCOI counterpart (Figure 9). Consequently, the increased surface roughness generated by the addition of 10% DCOI may have contributed to the similar biofilm coverages seen for polymer films of 5% and 10% DCOI (Figure 7).

## Conclusion

The addition of DCOI to PHB and PHBV provides a method to control the degradation rate of polyhydroxyalkanoates (PHAs) in a soil environment. The concentration of DCOI provided a means of tuning the onset and rate of film coverage by biofilm and subsequent degradation. The addition of the DCOI to the polymers had negligible effect on the physical properties such as temperature for melt and crystallisation. Additinoally, tensile strength was essentially unmodified due to the addition of the DCOI.

The onset of weight loss by degradation was proportional to the percent addition of DCOI with the optimal addition of 5% providing the longest delay in degradation onset for the least addition of DCOI.
